# The Contribution of Environmental Enrichment to Phenotypic Variation in Mice and Rats

**DOI:** 10.1523/ENEURO.0539-20.2021

**Published:** 2021-03-11

**Authors:** Amanda C. Kentner, Amanda V. Speno, Joanne Doucette, Ryland C. Roderick

**Affiliations:** 1 School of Arts and Sciences, Massachusetts College of Pharmacy and Health Sciences, Boston, MA 02115; 2 Library and Learning Resources, Massachusetts College of Pharmacy and Health Sciences, Boston, MA 02115

**Keywords:** animal welfare, coefficient of variation, environmental heterogeneity, phenotypic variability, sex differences, translation

## Abstract

The reproducibility and translation of neuroscience research is assumed to be undermined by introducing environmental complexity and heterogeneity. Rearing laboratory animals with minimal (if any) environmental stimulation is thought to control for biological variability but may not adequately test the robustness of our animal models. Standard laboratory housing is associated with reduced demonstrations of species typical behaviors and changes in neurophysiology that may impact the translation of research results. Modest increases in environmental enrichment (EE) mitigate against insults used to induce animal models of disease, directly calling into question the translatability of our work. This may in part underlie the disconnect between preclinical and clinical research findings. Enhancing environmental stimulation for our model organisms promotes ethological natural behaviors but may simultaneously increase phenotypic trait variability. To test this assumption, we conducted a systematic review and evaluated coefficients of variation (CVs) between EE and standard housed mice and rats. Given findings of suboptimal reporting of animal laboratory housing conditions, we also developed a methodological reporting table for enrichment use in neuroscience research. Our data show that animals housed in EE were not more variable than those in standard housing. Therefore, environmental heterogeneity introduced into the laboratory, in the form of enrichment, does not compromise data integrity. Overall, human life is complicated, and by embracing such nuanced complexity into our laboratories, we may paradoxically improve on the rigor and reproducibility of our research.

## Significance Statement

Environmental complexity is thought to increase phenotypic variability, undermining research translation. We conducted a systematic review and compared coefficients of variation (CVs) between environmentally enriched and standard housed laboratory animals. Despite there being no differences in variability across several phenotypic traits, there are stark contrasts in the display of ethological natural behaviors between these housing conditions. Environmental enrichment (EE) is recognized as being beneficial for animal welfare and mitigates against insults used to induce animal models of disease. Standard laboratory cages are instead recognized as being impoverished and “unnatural.” From these observations, it is apparent that our current “gold standard” caging system is not a true control condition as it does not adequately test the robustness of our animal models.

## Introduction

Contributions to phenotypic variation are thought to derive not only from genotype but from multiple environmental factors that range from feeding and microbiology, to variables as seemingly simple as housing condition. In experimental research, scientists attempt to control factors presumed to have an impact on biological variation and consequently the reproducibility of their data. One way to control for phenotypic variability in the laboratory is to standardize animal caging systems and limit environmental complexity. Environmental enrichment (EE) is one form of complexity that includes physical, sensory, cognitive, and/or social stimulation which provides an enhanced living experience to laboratory animals, relative to standard housing conditions. The use of EE has become prominent in neuroscience, because of substantial evidence that EE influences structural and functional changes in the brain, in addition to engendering enduring effects on behavior (Nithianantharajah and Hannan, 2006; Kempermann, 2019). Provisioning supplementary resources to animals not only maintains their welfare but promotes more naturalistic species typical behavioral repertoires (Bloomsmith et al., 2018). Moreover, this enhanced rearing condition has been used to study the mitigative potential of the environment in a variety of animal disease models (Nithianantharajah and Hannan, 2006).

Regardless of the purpose of its use, there are questions about potential within-experiment and between-experiment variability that may accompany the addition of environmental complexity to animal laboratory cages (Toth et al., 2011; Bayne and Würbel, 2014; Toth, 2015; Kempermann, 2019; Grimm, 2018; Sparling et al., 2020). It is thought that the diverse phenotypes promoted by EE may lead to data variation within a study. Moreover, the variety in enrichment protocols used may create data variability between studies and laboratories, compromising data reproducibility. Together, these concerns foster arguments to maintain barren cages as the “gold” standard housing condition (Bayne and Würbel, 2014; Voelkl et al., 2020). Importantly, similar justifications (of increased variation) have been used to support the exclusion of studying females in research, because of hormonal fluctuations across the reproductive cycle. However scientific evidence has since shown this perspective to be incorrect (Becker et al., 2016; Beery, 2018).

Given the shifting attention of the scientific community to the topic of rigor and reproducibility (Toth, 2015; Voelkl et al., 2020), this is the perfect time to reconsider our assumptions about variation because of environmental complexity. Standardization of the environment intuitively falls in line with the scientific method. Parsing out contributors of extraneous variation [phenotype (*P*) = gene × environmental interactions (*G × E*)] is thought to increase statistical power and reproducibility between experiments. On the other hand, such standardization leads to homogeneity in a population and may undermine the robustness of the potential treatment being studied (Kentner et al., 2018; for excellent recent review, see Voelkl et al., 2020), a crucial concern given the disconnect between preclinical and clinical research outcomes (Berk, 2012; Hyman, 2012; Munos, 2013).

Still, to control for potential variability, efforts to standardize the environment continues. These efforts have been complicated by varying definitions of what is enriching to animals of each species, strain, and sex (Simpson and Kelly, 2011; Toth et al., 2011; Toth, 2015), even for standard laboratory housing where only minimal EE is recommended or required. Moreover, a lack of reporting on what types of enrichment protocols are used (e.g., shelters, nesting materials, cage mates, music, food/treats; Toth, 2015) make this task even more difficult. Overall, the differential implementation of EE in experimental design has provoked discussion over the inconsistent definitions and reporting methodology of enrichment use in the neuroscience literature, and whether standardization and minimization of laboratory caging is necessary to prevent further extraneous biological variation (Bayne and Würbel, 2014; Toth, 2015).

Outside of theoretical debates, data on whether EE contributes to the replication crisis, by increasing phenotypic variability and undermining research findings, is mixed (Walsh and Cummins, 1979; Wolfer et al., 2004; Würbel, 2007; Toth et al., 2011; Toth, 2015) and concerns about its use persist (Grimm, 2018). Recently, there has been a call to action suggesting that the question of biological variation and its impact on rigor and reproducibility be extended to the diversification of environmental conditions or “controlled heterogenization” (Voelkl et al., 2020). For example, diversification may be implemented by using different sexes, animal strains, ages, and even housing conditions (e.g., EE) within a study. One way to address the question of variability because of the implementation of EE is to use the methods of others who have conducted large scale evaluations comparing between male and female animals (Becker et al., 2016) and inbred versus outbred strains of mice (Tuttle et al., 2018). Indeed, it has been noted that the EE literature has typically focused on mean ($\bar{x}$) differences between groups, rather than evaluating whether EE increases variability specifically (Kempermann, 2019). Of the small subset that have studied variation directly (Wolfer et al., 2004; Würbel, 2007; André et al., 2018) they have so far focused on mice and on a limited number of strains within the confines of their own experiments. To our knowledge, there has been no systematic literature-wide evaluation of multiple traits comparing EE to standard housed groups across species.

## Materials and Methods

To evaluate whether EE housed rats or mice display increased phenotypic variability in neuroscience research, we conducted a systematic review and compared the coefficient of variation (CV), a measure of trait-specific variability, extracted from data where EE animals of either sex were directly compared with a standard (control) housed condition on the same trait. First, to determine the general scientific interest in EE protocols, the proportion of articles published each year, using the search term “environmental enrichment” was identified in PubMed (Sperr, 2016).

### Search strategy

**Figure 1.**
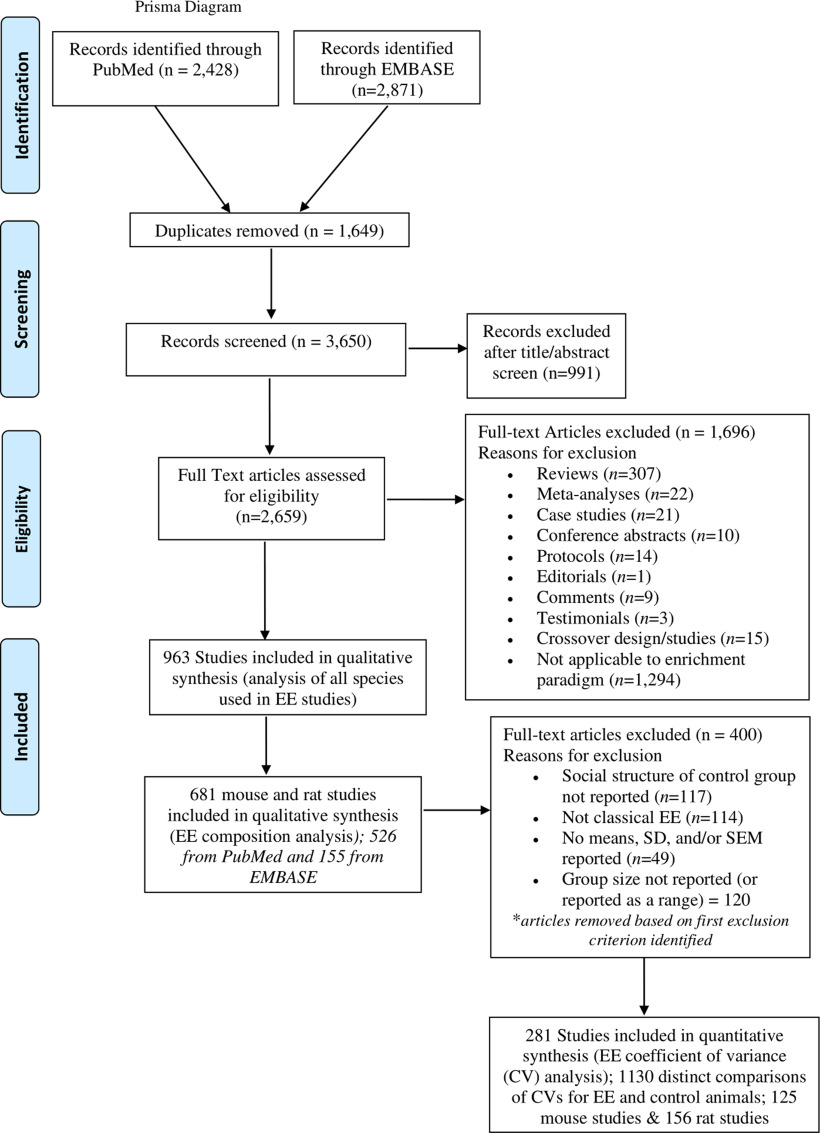
Prisma flow diagram.

Both PubMed and EMBASE were searched from the period of January first, 2013 to September 5, 2018, the date when these searches were initiated. The period evaluated is comparable to other important systematic reviews that assessed phenotypic variability (Becker et al., 2016). We used the search terms (1) EE AND (2) electrophysiology OR (3) brain OR (4) behavior OR (5) “nervous system physiological phenomena,” which yielded 3650 articles (Fig. 1).

### Study selection

**Figure 2.**
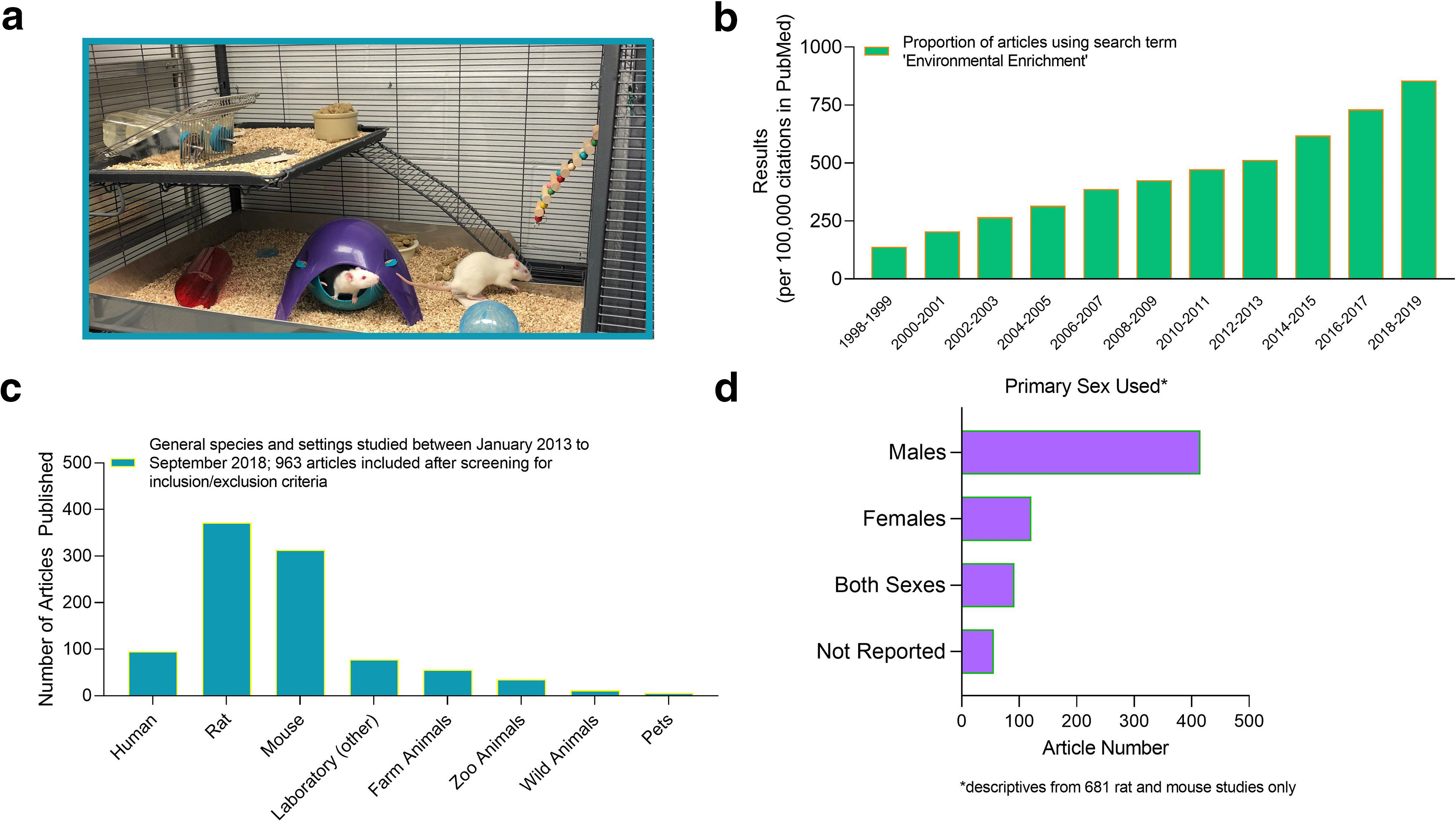
Descriptive analysis of common EE use in research. ***a***, Picture of a classic EE cage set-up for rodents. ***b***, Proportion per 100,000 citations of PubMed articles returned when searching “environmental enrichment.” Graph depicts articles published between 1998 and 2019. It includes both primary and secondary sources (an update from Simpson and Kelly, 2011). Graphs depict the (***c***) general species and settings and (***d***) primary sex studied using EE between January 2013 and September 2018. The selected articles used in this study are primarily from the areas of neuroscience and animal welfare (Extended Data Figs. 2-1, 2-2).

After duplicates were removed, evaluators independently identified studies eligible for inclusion in a 2-step process. First, we conducted an abstract and title search. If insufficient details were provided in the titles and abstracts, then the study was selected for full text review. Eligibility was based on (1) article relevance to the subject matter of interest (EE), (2) studies using any animal species including humans, (3) observational and experimental studies, and (4) English-written articles only. Exclusion criteria consisted of reviews, meta-analyses, case studies, conference abstracts, protocols, editorials, comments, and non-English articles. Overall, the articles included in this systematic review were primarily from the fields of neuroscience and animal welfare (see Fig. 2; Extended Data Figs. 2-1, 2-2).

10.1523/ENEURO.0539-20.2021.f2-1 Extended Data Figure 2-1 List of PubMed references used. Download Figure 2-1, DOCX file.

10.1523/ENEURO.0539-20.2021.f2-2 Extended Data Figure 2-2 List of EMBASE references used. Download Figure 2-2, DOCX file.

### Data extraction

Of the 963 articles identified as using EE in any species, a subset of 681 articles were identified as using mice or rats and were further evaluated on their use of several methodological variables including sex, types of enrichment devices employed, in addition to social structure of the EE group and composition of the control conditions used (e.g., running wheel, isolated, social/group housing). Phenotypic variability was also evaluated on the rat and mouse studies identified as using traditional EE caging systems (Fig. 2*A*). For these analyses, 281 studies were evaluated based on meeting the inclusion criteria of providing means and standard deviations (or standard errors) that could be extracted from the article, and sample sizes for at least one EE and one control group (Fig. 1). We also identified whether EE and control groups were naive or “treated/manipulated” (e.g., drug treated, knockout models, surgery etc.). Studies with parental exposure to EE were excluded to control for potential confounds of parental care (Connors et al., 2015), as were studies where it was unclear whether control animals were singly or socially housed. To avoid oversampling (Tuttle et al., 2018), we limited data collection to the first three reported measures where data and error bars were clearly legible. Each measure was categorized similarly to how others had done previously (Becker et al., 2016; Tuttle et al., 2018) by using behavior/CNS, behavior/other, anatomy, immune system function, organ function, molecules, and electrophysiology as traits. Generally, the behavior/CNS category included measures where animals demonstrated some type of learning, discrimination, or what could be considered more complex sequences of behaviors. Examples from our dataset include time spent with a novel object or social conspecific, sniffing duration, and duration of social contact (e.g., discrimination and preference tests). Number of lever responses, conditioned place preference scores, latency to locate a platform in the Morris water maze, % fear generalization, % freezing time, % sucrose preference, number of reference memory errors etc. were also included in this category. In contrast, the behavior/other category represented measures such as time spent in the center of the open field, frequency of crossings into the open center or periphery, and distance traveled in the open field. Anatomy included measures like the length or volume of brain regions (e.g., dendritic length, corpus callosum thickness). Immune system function as a category included measures such as flow cytometric analysis of CD40 on peritoneal macrophage, tumor volume or weight. We also placed plasma cytokine levels into this category. The organ function category included heart rate, changes in arterial blood P02, PC02, and pH, as well as fasting blood glucose levels. Molecules included any other measures of protein or mRNA, for example. These latter measures were primarily localized to the brain in our dataset.

In total, there were 1130 direct comparisons of CVs between EE and control animals included here (618 naive pair comparisons and 512 manipulated/treated pair comparisons; Fig. 1). The number of articles included, and direct comparisons made, in our analyses surpassed other excellent systematic reviews evaluating phenotypic variability (Becker et al., 2016; Tuttle et al., 2018). Therefore, we have an adequate sample size to make appropriate conclusions. Data were extracted from graphs provided on digital PDF articles (using https://rhig.physics.yale.edu/∼ullrich/software/xyscan/), or directly from tables (Tuttle et al., 2018). Graphical data extractions were performed by two trained researchers. Inter-rater reliability was assessed, and Pearson *r* correlation was determined to range from 0.912 to 0.997.

### Statistical analyses

CVs were calculated as standard deviation divided by the mean and compared using paired *t* tests (for individual trait evaluations), or ANOVA (for multiple trait evaluations). Pairwise comparisons were done using the Tukey’s multiple comparisons test (Howell, 2001; Becker et al., 2016). The partial η^2^ is also reported as an index of effect size for the ANOVAs (the range of values being 0.02 = small effect, 0.13 = moderate effect, 0.26 = large effect; Miles and Shevlin, 2001). To determine whether the distribution of variation differed by environmental complexity, we calculated EE to control ratios of *CV* = [(*CV_EE_)/(CV_EE_ + CV_control_*)]. CV ratios for each trait were tested as a function of housing complexity against the theoretical mean 0.5 by *t* test (Becker et al., 2016; Beery, 2018). Data were considered significant if *p* < 0.05.

## Results

Using the term “environmental enrichment,” we identified the proportion of articles indexed in PubMed each year from 1998 to 2019 (Sperr, 2016). One report has previously evaluated the number of articles published from 1960 to 2009 (Simpson and Kelly, 2011). In this work, it was demonstrated that an increased interest in EE emerged between 1990–1999 and 2000–2009. Here, we provide a replication and extension of those data from 1998 until 2019. Our search, including both review and empirical research articles, highlights a continuation of the increasing interest on this topic, relative to the number of total articles published (Fig. 2*B*).

**Figure 3.**
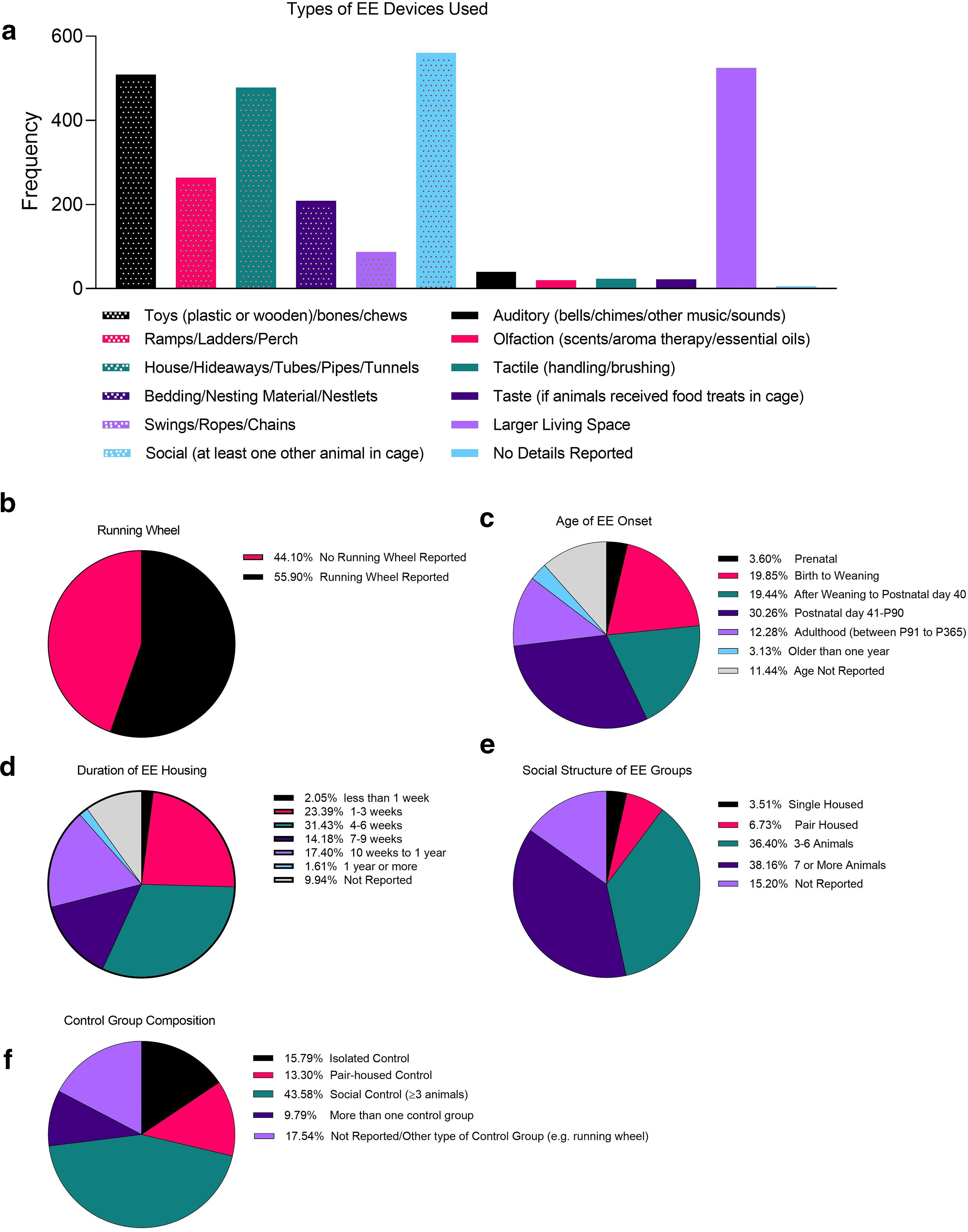
Descriptive analysis of common EE methodology. All descriptive data are from rat and mice studies where animals are housed in a classic EE design. Data outline the (***a***) frequency of types of EE devices used, in addition to the percentage of EE studies using (***b***) running wheels, or a particular (***c***) age of EE onset, (***d***) duration of EE housing, and general/social structure of (***e***) EE and (***f***) control groups. Data derived from a total of 681 research articles published between January 2013 and September 2018.

The results of our analyses demonstrate patterns of experimental biases, specifically a heavy reliance on the use of rats and mice over other laboratory species (Fig. 2*C*), and the continued exclusion of females in EE research (Fig. 2*D*; Simpson and Kelly, 2011). Our findings also show a range in the definition of EE used across laboratories in that the frequency of enrichment types, timing, and the social structures implemented varied widely (Fig. 3*A–F*). The use of toys (including plastic or wooden), bones/chews, house hideaways, or tubes/pipes and tunnels, in addition to a larger cage space and social conspecifics were more frequently used in the enrichment housing conditions. Supplementary bedding/nesting materials and ramps/ladders or perches were less commonly used, as were swings, ropes and chains (Fig. 3*A*).

One issue that arose was a significant lack of reporting on several variables. This prompted us to develop a reporting table for describing key aspects of enrichment use in research (Table 1), following suit with other initiatives to improve on animal model reporting (Kentner et al., 2019). As part of this table, we suggest authors report whether they are providing EE animals with manufactured/artificial enrichment devices or more natural stimuli as there are differences in animal phenotypes depending on these devices (Hess et al., 2008; Lambert et al., 2015).

**Table 1:** Environmental enrichment reporting guidelines checklist. The recommended use of this reporting form is to fill it out and include it as supplemental material for each of your laboratory’s environmental enrichment research publications. This document can also be used as a guide for including details of cage enrichment for studies using only standard laboratory housing. If there are difficulties using/adapting this form, please contact one of the corresponding authors to request a copy.

Reporting item	Description
EE cage size (L × W × H) in cm	
Control cage size (L × W × H) in cm	
Material of EE cages	
Material of control cages	
Type of EE caging system (e.g., open top/individually ventilated)	
Type of control caging system (e.g., open top/individually ventilated)	
Number and sex of animals housed in each EE cage	
Number and sex of animals housed in each control cage	
Enrichment devices (e.g., toys, shelters, ramps, running wheels, perches, or different levels/shelves) in EE cages.What type of materials were the devices made of (e.g., wood, plastic, are they manufactured/artificial, or are the natural stimuli?). How many enrichment devices were typically in the cage?	
Enrichment devices (e.g., toys, shelters, ramps, running wheels, perches, or different levels/shelves) in control cages. What type of materials were the devices made of (e.g., wood, plastic, are they manufactured/artificial, or are the natural stimuli?). How many enrichment devices were typically in the cage?	
How frequently were enrichment devices changed in EE cages?	
How frequently were enrichment devices changed in control cages?	
Bedding type and other nesting materials in EE cages	
Bedding type and other nesting materials in control cages	
Age of animals when placed into EE housing	
Duration housed in EE cages	
Frequency of cage cleanings for EE animals	
Frequency of cage cleanings for control animals	
Were EE and control animals housed in the same room or in a different one?	
Specific number of EE and control animals used for each measure reported	
Other notes/comments (e.g., other types of enrichment given, for example, music/sounds in animal holding room, food treats)	

Using paired *t* tests, we found no differences between EE and standard housed mice or rats on CVs across traits (*p* > 0.05), regardless of control housing type (e.g., running wheel, isolated, social/group housing) or whether animals were naive or manipulated/treated (e.g., drug treated, knockout models, surgery). Therefore, we collapsed and analyzed both species together. When species were combined, the treated/manipulated social/group housed controls (0.65 ± 0.073) were more variable than their manipulated/treated EE counterparts (0.59 ± 0.050; *t*_(46)_ = 2.211, *p* = 0.032) on the “behavior other” trait only. Isolated control animals (0.24 ± 0.079) had higher CVs than treated/manipulated EE animals on the anatomy trait (0.019 ± 0.072; *t*_(4)_ = 4.720, *p* = 0.009). However, for the anatomy trait the number of available comparisons between these two groups was not sufficiently powered (*n* = 5 comparisons based on three articles). In general, we did not find EE to increase trait variability compared with any control housing type in either naive or manipulated/treated animals (*p* > 0.05).

To increase the power in our analyses, we collapsed the control group types together and analyzed across species and traits, both separately and together. Again, we found that EE does not make animals more variable than controls (*p* > 0.05; Fig. 4*A–D*; Extended Data Figs. 4-1, 4-2, 4-3, 4-4, 4-5, 4-6, 4-7, 4-8, 4-9, 4-10, 4-11, 4-12, 4-13, 4-14, 4-15, 4-16, 4-17, 4-18, 4-19, 4-20, 4-21, 4-22, 4-23, 4-24, 4-25, 4-26). When species were combined, we found that controls were more variable (had higher CVs) than EE housed animals under treated/manipulated conditions. However, this was only found on the “overall behavior” (main effect of housing: *t*_(290)_ = 2.120, *p* = 0.035; control CV: 0.67 ± 0.06/EE CV: 0.56 ± 0.04) and “behavior other” traits (main effect of housing: *t*_(46)_ = 2.211, *p* = 0.032; control CV: 0.73 ± 0.07/EE CV: 0.60 ± 0.05, based on 21 articles; Fig. 4 *B*,*D*; Extended Data Fig. 4-4).

**Figure 4.**
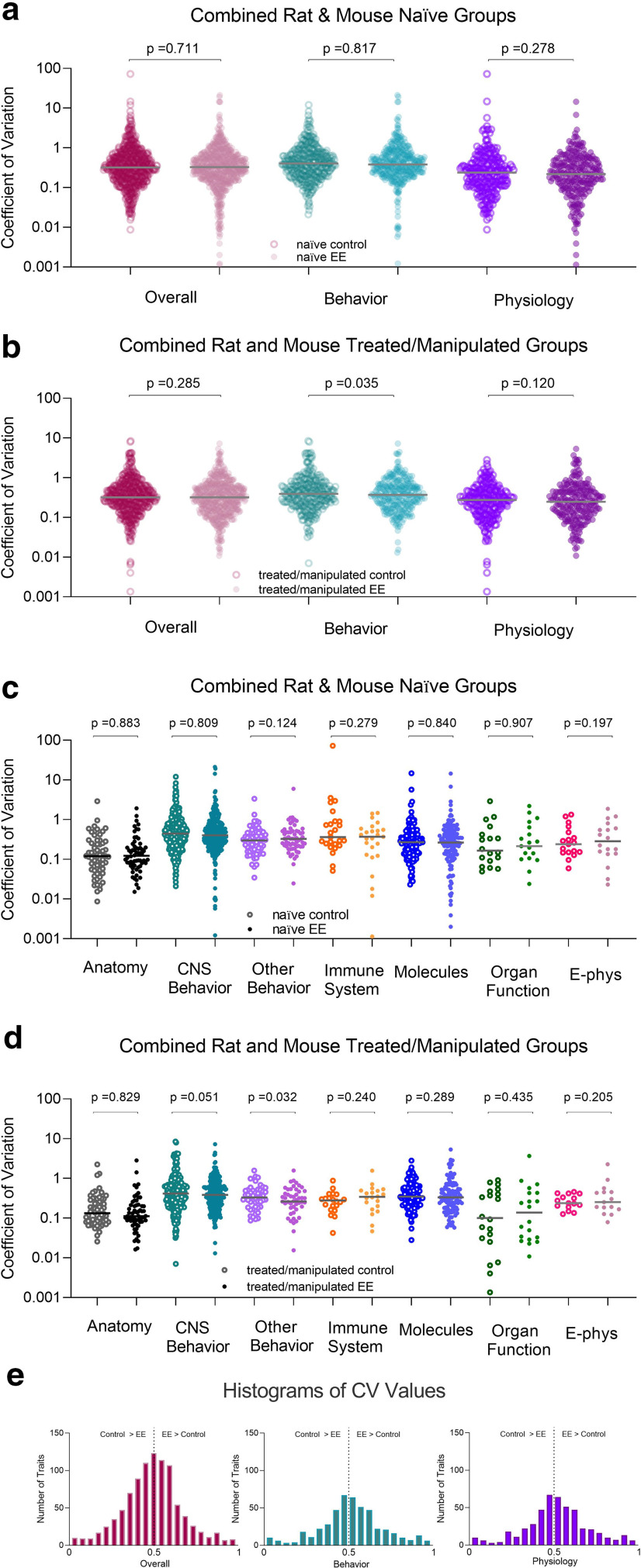
“Coefficients of variation” for all studies where control and EE mice or rats were directly compared. All data presented as the overall trait variance and further separated into subcategories of behavior and physiology as well as seven specific trait measures for (***a***, ***c***) naive/untreated and (***b***, ***d***) treated/manipulated animals (mean ± SEM). Each data point represents a single control or EE measure from a single experiment along with the mean for each respective trait. Coefficient of variance was calculated as the standard deviation divided by the mean for each data point. ***e***, Histogram of distribution of CV ratios (EE CV/EE CV + control CV) collapsed across naive and treated/manipulated mice and rats. To determine whether the variance from the mean was normally distributed for the different traits, we evaluated the CV ratios (*p* values from Extended Data Figs. 4-1, 4-2, 4-3, 4-4, 4-5, 4-6, 4-7, 4-8, 4-9, 4-10, 4-11, 4-12, 4-13, 4-14, 4-15, 4-16, 4-17, 4-18, 4-19, 4-20, 4-21, 4-22, 4-23, 4-24, 4-25, 4-26). A value of 0.5 (black dotted line) indicates that EE and control animals are similar. Values to the right suggest that EE animals are more variable than controls.

10.1523/ENEURO.0539-20.2021.f4-1 Extended Data Figure 4-1 Pairwise comparisons for naive controls and naive enriched rats and mice in which all behavior, physiology, and anatomy traits are combined. Download Figure 4-1, DOCX file.

10.1523/ENEURO.0539-20.2021.f4-2 Extended Data Figure 4-2 Pairwise comparisons for treated/manipulated controls and treated/manipulated enriched rats and mice in which all behavior, physiology, and anatomy traits are combined. Download Figure 4-2, DOCX file.

10.1523/ENEURO.0539-20.2021.f4-3 Extended Data Figure 4-3 Pairwise comparisons for naive controls and naive enriched rats and mice by each individual trait. Download Figure 4-3, DOCX file.

10.1523/ENEURO.0539-20.2021.f4-4 Extended Data Figure 4-4 Pairwise comparisons for treated/manipulated controls and treated/manipulated enriched rats and mice by each individual trait. Download Figure 4-4, DOCX file.

10.1523/ENEURO.0539-20.2021.f4-5 Extended Data Figure 4-5 Pairwise comparisons for naive control and naive enriched rats in which all behavior, physiology, and anatomy traits are combined. Download Figure 4-5, DOCX file.

10.1523/ENEURO.0539-20.2021.f4-6 Extended Data Figure 4-6 Pairwise comparisons for treated/manipulated control and treated/manipulated enriched rats in which all behavior, physiology, and anatomy traits are combined. Download Figure 4-6, DOCX file.

10.1523/ENEURO.0539-20.2021.f4-7 Extended Data Figure 4-7 Pairwise comparisons for naive controls and naive enriched rats by each individual trait. Download Figure 4-7, DOCX file.

10.1523/ENEURO.0539-20.2021.f4-8 Extended Data Figure 4-8 Pairwise comparisons for treated/manipulated controls and treated/manipulated enriched rats by each individual trait. Download Figure 4-8, DOCX file.

10.1523/ENEURO.0539-20.2021.f4-9 Extended Data Figure 4-9 Pairwise comparisons for naive controls and naive enriched mice in which all behavior, physiology, and anatomy traits are combined. Download Figure 4-9, DOCX file.

10.1523/ENEURO.0539-20.2021.f4-10 Extended Data Figure 4-10 Pairwise comparisons for treated/manipulated controls and treated/manipulated enriched mice in which all behavior, physiology, and anatomy traits are combined. Download Figure 4-10, DOCX file.

10.1523/ENEURO.0539-20.2021.f4-11 Extended Data Figure 4-11 Pairwise comparisons for naive controls and naive enriched mice by each individual trait. Download Figure 4-11, DOCX file.

10.1523/ENEURO.0539-20.2021.f4-12 Extended Data Figure 4-12 Pairwise comparisons for treated/manipulated controls and treated/manipulated enriched mice by each individual trait. Download Figure 4-12, DOCX file.

There were no main effects of housing, nor significant housing by trait interactions on the two-way ANOVAs (*p* > 0.05, Fig. 4; Extended Data Fig. 4-13). However, there were significant main effects of trait, indicating that “behavior” was more variable than “anatomic” traits for both rats (main effect of trait: *F*_(5,542)_ = 4.015, *p* = 0.001, η^2^ = 0.036; Tukey HSD: *p* = 0.004) and mice (main effect of trait: *F*_(6,460)_ = 4.953, *p* = 0.0001, η^2^ = 0.057; Tukey HSD: *p* = 0.001; Fig. 4; Extended Data Fig. 4-13). Of special note, partial η^2^ values were indicative of small effect sizes for these comparisons.

10.1523/ENEURO.0539-20.2021.f4-13 Extended Data Figure 4-13 Two-way ANOVAs comparing multiple traits (all behavior, physiology, anatomy) by housing condition (EE, standard housing) for the independent variable CV. Data presented for both rats and mice combined and separately. Download Figure 4-13, DOCX file.

10.1523/ENEURO.0539-20.2021.f4-14 Extended Data Figure 4-14 CV distributions for naive standard housed (controls) and naive EE rats and mice combined with treated/manipulated controls and treated/manipulated EE rats and mice in which all behavior, physiology, and anatomy traits are combined. CV ratios were used to determine whether the distribution of variation differed by environmental complexity. Calculated EE to control ratios of *CV* = [(*CV_EE_)/(CV_EE_ + CV_control_*)]. CV ratios tested as a function of housing complexity against the theoretical mean of 0.5 by a one-sample *t* test. Download Figure 4-14, DOCX file.

10.1523/ENEURO.0539-20.2021.f4-15 Extended Data Figure 4-15 CV distributions for naive standard housed (controls) and naive EE rats and mice in which all behavior, physiology, and anatomy traits are combined. CV ratios were used to determine whether the distribution of variation differed by environmental complexity. Calculated EE to control ratios of *CV* = [(*CV_EE_)/(CV_EE_ + CV_control_*)]. CV ratios tested as a function of housing complexity against the theoretical mean of 0.5 by a one-sample *t* test. Download Figure 4-15, DOCX file.

10.1523/ENEURO.0539-20.2021.f4-16 Extended Data Figure 4-16 CV distributions for treated/manipulated standard housed (controls) and treated/manipulated EE rats and mice in which all behavior, physiology, and anatomy traits are combined. CV ratios were used to determine whether the distribution of variation differed by environmental complexity. Calculated EE to control ratios of *CV* = [(*CV_EE_)/(CV_EE_ + CV_control_*)]. CV ratios tested as a function of housing complexity against the theoretical mean of 0.5 by a one-sample *t* test. Download Figure 4-16, DOCX file.

10.1523/ENEURO.0539-20.2021.f4-17 Extended Data Figure 4-17 CV distributions for naive standard housed (controls) and naive EE rats and mice by each individual trait. CV ratios were used to determine whether the distribution of variation differed by environmental complexity. Calculated EE to control ratios of *CV* = [(*CV_EE_)/(CV_EE_ + CV_control_*)]. CV ratios tested as a function of housing complexity against the theoretical mean of 0.5 by a one-sample *t* test. Download Figure 4-17, DOCX file.

10.1523/ENEURO.0539-20.2021.f4-18 Extended Data Figure 4-18 CV distributions for treated/manipulated standard housed (controls) and treated/manipulated EE rats and mice by each individual trait. CV ratios were used to determine whether the distribution of variation differed by environmental complexity. Calculated EE to control ratios of *CV* = [(*CV_EE_)/(CV_EE_ + CV_control_*)]. CV ratios tested as a function of housing complexity against the theoretical mean of 0.5 by a one-sample *t* test. Download Figure 4-18, DOCX file.

10.1523/ENEURO.0539-20.2021.f4-19 Extended Data Figure 4-19 CV distributions for naive standard housed (controls) and naive EE rats in which all behavior, physiology, and anatomy traits are combined. CV ratios were used to determine whether the distribution of variation differed by environmental complexity. Calculated EE to control ratios of *CV* = [(*CV_EE_)/(CV_EE_ + CV_control_*)]. CV ratios tested as a function of housing complexity against the theoretical mean of 0.5 by a one-sample *t* test. Download Figure 4-19, DOCX file.

10.1523/ENEURO.0539-20.2021.f4-20 Extended Data Figure 4-20 CV distributions for treated/manipulated standard housed (controls) and treated/manipulated EE rats in which all behavior, physiology, and anatomy traits are combined. CV ratios were used to determine whether the distribution of variation differed by environmental complexity. Calculated EE to control ratios of *CV* = [(*CV_EE_)/(CV_EE_ + CV_control_*)]. CV ratios tested as a function of housing complexity against the theoretical mean of 0.5 by a one-sample *t* test. Download Figure 4-20, DOCX file.

10.1523/ENEURO.0539-20.2021.f4-21 Extended Data Figure 4-21 CV distributions for naive standard housed (controls) and naive EE rats by each individual trait. CV ratios were used to determine whether the distribution of variation differed by environmental complexity. Calculated EE to control ratios of *CV* = [(*CV_EE_)/(CV_EE_ + CV_control_*)]. CV ratios tested as a function of housing complexity against the theoretical mean of 0.5 by a one-sample *t* test. Download Figure 4-21, DOCX file.

10.1523/ENEURO.0539-20.2021.f4-22 Extended Data Figure 4-22 CV distributions for treated/manipulated standard housed (controls) and treated/manipulated EE rats by each individual trait. CV ratios were used to determine whether the distribution of variation differed by environmental complexity. Calculated EE to control ratios of *CV* = [(*CV_EE_)/(CV_EE_ + CV_control_*)]. CV ratios tested as a function of housing complexity against the theoretical mean of 0.5 by a one-sample *t* test. Download Figure 4-22, DOCX file.

10.1523/ENEURO.0539-20.2021.f4-23 Extended Data Figure 4-23 CV distributions for naive standard housed (controls) and naive EE mice in which all behavior, physiology, and anatomy traits are combined. CV ratios were used to determine whether the distribution of variation differed by environmental complexity. Calculated EE to control ratios of *CV* = [(*CV_EE_)/(CV_EE_ + CV_control_*)]. CV ratios tested as a function of housing complexity against the theoretical mean of 0.5 by a one-sample *t* test. Download Figure 4-23, DOCX file.

10.1523/ENEURO.0539-20.2021.f4-24 Extended Data Figure 4-24 CV distributions for treated/manipulated standard housed (controls) and treated/manipulated EE mice in which all behavior, physiology, and anatomy traits are combined. CV ratios were used to determine whether the distribution of variation differed by environmental complexity. Calculated EE to control ratios of *CV* = [(*CV_EE_)/(CV_EE_ + CV_control_*)]. CV ratios tested as a function of housing complexity against the theoretical mean of 0.5 by a one-sample *t* test. Download Figure 4-24, DOCX file.

10.1523/ENEURO.0539-20.2021.f4-25 Extended Data Figure 4-25 CV distributions for naive standard housed (controls) and naive EE mice by each individual trait. CV ratios were used to determine whether the distribution of variation differed by environmental complexity. Calculated EE to control ratios of *CV* = [(*CV_EE_)/(CV_EE_ + CV_control_*)]. CV ratios tested as a function of housing complexity against the theoretical mean of 0.5 by a one-sample *t* test. Download Figure 4-25, DOCX file.

10.1523/ENEURO.0539-20.2021.f4-26 Extended Data Figure 4-26 CV distributions for treated/manipulated standard housed (controls) and treated/manipulated EE mice by each individual trait. CV ratios were used to determine whether the distribution of variation differed by environmental complexity. Calculated EE to control ratios of *CV* = [(*CV_EE_)/(CV_EE_ + CV_control_*)]. CV ratios tested as a function of housing complexity against the theoretical mean of 0.5 by a one-sample *t* test. Download Figure 4-26, DOCX file.

Although the inclusion of female animals was demonstrably lower than males to be able to make adequately powered comparisons on many traits (Fig. 2*D*), we conducted some preliminary sex difference analyses. Our subanalyses revealed that naive male EE rats (0.60 ± 0.10) had higher *CVs* than their naive social/group housed controls (0.39 ± 0.18; *t*_(32)_ = −2.266, *p* = 0.030, based on 18 articles) on the “behavior other” trait, but were not more variable on any other trait (*p* > 0.05). There were no further differences in variability between EE and control animals across any combination of sex, strain, control type, or naive versus treated/manipulated animals.

When comparing CV ratios, the data did not support the premise that environmental complexity increases variability in neuroscience research (*p* > 0.05; Fig. 4*E*; Extended Data Figs. 4-14, 4-15, 4-16, 4-17, 4-18, 4-19, 4-20, 4-21, 4-22, 4-23, 4-24, 4-25, 4-26).

## Discussion

Our findings should resonate well with neuroscientists who would like to increase complexity in laboratory caging systems, promoting more naturalistic species typical behaviors and brain functioning, but who have been concerned about compromising data integrity and their control over environmental conditions. This should be especially salient given that lack of enrichment in laboratory cages leads to suppression of behavioral repertoires, increased stereotypies, and a reduction of general activity level, even during an animals’ active phase (Hurst et al., 1997). Indeed, deprivation in the environment is known to impact the structure and functioning of the brain, affecting cognition and behavior (Lahvis, 2017; McLaughlin et al., 2017). This underscores the view that our current standard laboratory housing condition is not a true control condition. Cage enrichment is recommended in the Guide for the Care and Use of Laboratory Animals (National Research Council, 2011), and for standard housed rodents typically takes the form of sanitizable polyvinyl chloride (PVC) tubes, a chew bone, or a piece of nesting material. If the animal is lucky, they may receive a combination of two or three pieces of these enrichment devices. To be frank, the composition of this housing condition needs a major renovation. Seldom do these cage enrichment objects change across the course of the study; novelty and increased stimulation are luxuries afforded to animals reared in classic EE (see Fig. 2*A*). This latter housing condition is rarely used as a standard in the laboratory; when employed, EE is usually for the purpose of exploring mechanisms underlying neural plasticity, or to mitigate some type of toxic insult (Nithianantharajah and Hannan, 2006). The availability of resources is a major restriction to increasing stimulation in the animal laboratory. It will require a change in the mindsets of institutions, scientists, and funding bodies to make this housing condition, or an adapted version, the new “gold standard.” Some solutions to address cost, physical space, as well as personnel constraints to implementing higher levels of enrichment have been discussed elsewhere (Kentner et al., 2018) and are outlined below. Still, the direction of funds to establish more complex housing conditions for laboratory animals should be part of the movement to improve scientific rigor and reproducibility.

Another important hurdle to the implementation of EE is concerns about phenotypic variability because of increased heterogeneity. While we identified some increased variability in naive male EE rats on measures such as distance traveled and open field, most studies evaluated used some type of experimental treatment/manipulation which did not affect phenotypic variability on any trait. Moreover, others have reported no differences in variability on these types of measures, when associated with EE use, at least in mice (Wolfer et al., 2004; Würbel, 2007; André et al., 2018). In general, this species and sex specific effect suggests that researchers may need to identify the appropriate EE devices to use for male rats in some experimental designs, to resolve potential issues in variability. Notably, we observed increased phenotypic variability on the “overall behavior” and “behavior other” traits in control housed animals. Therefore, complex housing does not make animals any more variable in comparison to standard laboratory housing. One consideration with respect to our data are that a larger proportion of our analyses were behavioral measures, versus cellular or molecular. However, these latter measures were also equally unaffected by housing condition. Still, our interpretations are limited by the fact that we summarized many studies and that these overall findings may not be applicable to individual experiments. Moreover, factors such as age of EE onset, animal age at endpoint evaluation, strain differences, as well as other species differences are important contributions to EE that may affect our interpretations. Another potential contributor to the shaping of phenotype could be the shared experiences in EE, resulting in within-group differences. Individual animals influence their environment, and each other, affecting phenotypes and preventing full control of the environment. Therefore, EE could be considered not just as *P *=* G × E*, but as *G ×* (*E_shared_ + E_nonshared_*) (for excellent review, see Kempermann, 2019). This equation is also relevant to pair and grouped standard laboratory cage housing, which do not increase phenotypic trait variability (Becker et al., 2016), similarly to what we show here with more naturalistic settings.

Together, the main complaints against the implementation of EE have been about feasibility and associated financial costs, in addition to arguments of increased phenotypic variability as a result of modeling more naturalistic settings in the laboratory environment (Grimm, 2018). However, EE may not need to be extravagant or require larger caging systems and space but may be as simple as regularly changing enrichment devices (Kentner et al., 2018). Notably, investigators often group house their animals to reduce stress (Hurst et al., 1997), and consequentially save on laboratory caging costs. One option is to use bigger cages that take up the same space/area as multiple smaller cages. Animals can then be grouped together in larger colonies. While this can serve to increase social enrichment, its implementation must also keep the needs of each species and sex in mind. For example, issues of social hierarchy and dominance are more likely to occur in males of some species and social stress experiences can greatly affect overall health and disposition (Larrieu et al., 2017; Zhou et al., 2018; Beery et al., 2020). Species such as CD-1 mice will become territorial when enrichment devices are introduced into the environment, disrupting their established hierarchy (McQuaid et al., 2012). These types of species may otherwise live cooperatively in a larger group when the social hierarchy is firmly established (McQuaid et al., 2012; for excellent review on groups and non-traditional housing models, see Beery et al., 2020). Importantly, most EE studies begin to offer increased stimulation at weaning, or shortly after puberty (see Fig. 3*C*). In at least some animal models, when higher levels of stimulation are the norm across the entire lifespan, versus being introduced after adolescence, fighting has been reported to be non-existent in both male and female mice and rats. This has allowed for the peaceful use of enrichment devices among these groups (Connors et al., 2014; Kentner et al., 2016; Zhao et al., 2021).

From a purely scientific perspective, EE can mitigate the effects of several experimental treatments and animal models of disease (Nithianantharajah and Hannan, 2006) and is often interpreted as a beneficial intervention (Sparling et al., 2020). However, this calls into question the external validity of these apparent context specific effects (Bernard, 2019; Manouze et al., 2019) and the robustness of our animal models; a clear example of fallacious reasoning (Bernard, 2020). Indeed, incorporating more environmental heterogeneity into neuroscience research, and testing our findings against such complexity, should increase the robustness of our experimental designs and the fidelity of biomedical treatments (Kentner et al., 2018; Voelkl et al., 2020), without compromising the underlying stability of data. Our study supports this idea given that traditional EE caging systems are dynamic environments where devices are being replaced or are changing location as animals interact and move them. Moreover, social experiences are varied for each animal. Specifically, experiences both between and within EE cages are unique, yet complex housing does not make animals any more variable compared with standard laboratory housed rats or mice. Importantly, the increased use of EE and improved robustness of experimental design should be less costly in the long run. This contrasts with a continued reliance on standard laboratory housing, which is clearly not a true control condition and appears to impede the translation of research results.

Going forward, it will be necessary to identify appropriate enrichment types for the species, sex, and age of the model organism of interest, in addition to the animal model/paradigm being used, and to accurately report their use (Simpson and Kelly, 2011; Toth, 2015; Kentner et al., 2018). Importantly, there are proposed methodologies for how to implement and account for such environmental variation (Voelkl et al., 2020). Overall, human life is complicated and by embracing such nuanced complexity into our laboratories we may paradoxically improve on the rigor and reproducibility of our research.

### Data availability

All data are available on request.

### Code availability

There is no code associated with this work.

## Synthesis

Reviewing Editor: Erica Glasper, University of Maryland Decisions are customarily a result of the Reviewing Editor and the peer reviewers coming together and discussing their recommendations until a consensus is reached. When revisions are invited, a fact-based synthesis statement explaining their decision and outlining what is needed to prepare a revision will be listed below. The following reviewer(s) agreed to reveal their identity: Kelly Lambert.

Kentner and colleagues raise important concern in this systematic review examining the importance of phenotypic variation in preclinical studies of environmental enrichment in rodents. The authors have thoughtfully evaluated 681 neuroscience-related papers with the goal of determining to what extent varied experiences of environmental enrichment contribute to greater variability across individuals.

Major Concerns:

1. Throughout the manuscript, it is suggested that a difference in variability (i.e., coefficient of variation) between control and enriched environments would compromise the translatable value of enriched housing protocols. If the enriched environment is indeed more ethologically and ecologically relevant, then any housing conditions that differ from those criteria would compromise the translational value of the results, regardless of how close the variability trends are to the "gold standard" (e.g., barren cages). If these impoverished conditions are viewed as unnatural, it becomes clear that this housing condition was never truly a "control" condition. This should be more strongly related to the reader.

2. The authors have approached this analysis from a broad point of view. This is both a strength and a weakness to the manuscript. The inclusion of many studies that likely reduces bias, raises questions as to what types of studies were included. The following questions emerge:

a. What was the primary field of the research group?

b. What were the exact endpoints examined?

The reviewers realize that it would be difficult to include these details, but the authors are encouraged to increase the discussion of the nature of these within the methods section.

3. The authors are encouraged to add titles to the Tables - this would help to increase more information about the subject and/or goal of the various studies.

a. Additionally, it is unclear how the categories of "behavior (non-CNS) and "other behavior" were defined. Providing more information will be informative to the reader.

b. More information should be added to Table 2 (the table that includes the information that was recorded for each article). Adding more information about the "enrichment devices" category considering that a distinction has been made between manufactured or artificial and more natural stimuli has been investigated (https://www.ncbi.nlm.nih.gov/pmc/articles/PMC4774049/). Did the authors systematically assess this variable? Was the number of enrichment devices in the cages considered?

4. The authors are cautioned that the language used to describe the results may be rather strong and may be a limitation. This should be noted as a limitation in the discussion section and framed in a way that readers understand that numerous studies are summarized together and that the statements may not hold for individual studies.

5. Greater information regarding "traits" should be added to the manuscript. Heading such as "anatomy," "CNS behavior," "molecules," etc do not provide the reader with enough information. Greater clarification could be gained by adding information regarding what specific endpoints are contributing to the observed effects.

6. The authors are encouraged to increase the discussion regarding hierarchy and dominance as the implications could be interesting. This is very meaningful when discussing sex differences, as males are more likely to form hierarchies than females.

7. The authors are encouraged to increase their discussion of cage enrichment, which is currently the prescribed veterinary guideline. This should be discussed in comparison to environmental enrichment. While this is not the goal of the present analysis, the differences between these comparisons should be noted.

8. More clarification about the following sentence on page 7 would be helpful to the reader: "For those who conduct studies in the field of EE directly, our findings suggest that more complex behavioral measures may be best evaluated over the use of open field test and distance traveled." Please provide more examples of "more complex behavioral measures" that can be provided as well as more information about how the authors came to this conclusion.

Minor Concerns:

1. The authors should add a brief discussion of other factors that may be contributing to observed effects, such as age, age of onset, and species/strain differences. While these are not the main experimental factors, they each have major on EE effects and should be noted as important factors in addition to sex and EE paradigm. This goes along with the limitations statement from #1.

2. Are the tables meant to be included in the paper or added as supplements? They seem more like supplemental material but appear to be for the main paper. Please clarify and also add legends to the tables to better describe the data being tabulated.

3. The authors do a good job of summarizing such questions regarding differential EE and CON designs in Figure 2. This is a big problem in EE research, and it is interesting to see the breakdown here. Is it possible to add some analyses focused on whether these variables have consistent impact on the variability results (i.e., could an ANCOVA be employed?). This may be beyond the scope of the manuscript; however, it would be beneficial to help answer questions surrounding whether differential environmental enrichment paradigms across labs negatively impact reproducibility.

4. Minor correction: On page 6, following "suite" should be changed to following "suit."
