# Supplementary material for: The Contribution of Environmental Enrichment to Phenotypic Variation in Mice and Rats
Source: eNeuro. 2021 Mar 11;8(2):ENEURO.0539-20.2021. doi: 10.1523/ENEURO.0539-20.2021 (PMC7986535; doi:10.1523/ENEURO.0539-20.2021)
Supplement: Extended Data Figure 4-2 — Pairwise comparisons for treated/manipulated controls and treated/manipulated enriched rats and mice in which all behavior, physiology, and anatomy traits are combined. Download Figure 4-2, DOCX file. [file enu-eN-NWR-0539-20-s05.docx]

**Extended Data Table 4-2**. Pairwise comparisons for treated/manipulated controls and treated/manipulated enriched rats and mice in which all behavior, physiology, and anatomy traits are combined.

| Description | Trait Category | Mean | Standard  Deviation | Standard Error | 95% confidence interval | | t | df | p-value  (two tailed) |
| --- | --- | --- | --- | --- | --- | --- | --- | --- | --- |
|  |  |  |  |  | Lower | Upper |  |  |  |
| Main effect of housing | all traits combined | .036 | .770 | .034 | -.030 | .103 | 1.071 | 513 | .285 |
