# Supplementary material for: The Contribution of Environmental Enrichment to Phenotypic Variation in Mice and Rats
Source: eNeuro. 2021 Mar 11;8(2):ENEURO.0539-20.2021. doi: 10.1523/ENEURO.0539-20.2021 (PMC7986535; doi:10.1523/ENEURO.0539-20.2021)
Supplement: Extended Data Figure 4-13 — Two-way ANOVAs comparing multiple traits (all behavior, physiology, anatomy) by housing condition (EE, standard housing) for the independent variable CV. Data presented for both rats and mice combined and separately. Download Figure 4-13, DOCX file. [file enu-eN-NWR-0539-20-s16.docx]

**Extended Data Table 4-13**. Two-way ANOVAs comparing multiple traits (all behavior, physiology, anatomy) by housing condition (environmental enrichment, standard housing) for the independent variable coefficient of variation (CV). Data presented for both rats and mice combined and separately.

| **Species** | **Housing** | **ANOVA** | **Tukey HSD** |
| --- | --- | --- | --- |
| **Rat and Mouse Data Combined** | *Naïve Controls and Naïve EE* | housing x trait: F(6, 1014) = 0.790, p = 0.643, ƞ^2^= 0.004  housing: F(1, 1014) = 0.349, p = 0.555, ƞ^2^= 0.0001  trait: F(6, 1014) = 7.336, p = 0.0001, ƞ^2^= 0.042 | *behavior (cns) vs behavior (other)*: p =0.017  *behavior (cns) vs anatomy*: p = 0.0001  *anatomy vs molecules*: p = 0.012 |
|  | *Treated/Manipulated Controls and Treated/Manipulated EE* | housing x trait: F(6, 1024) = 0.078, p =0.998, ƞ^2^=0.0001  housing: F(1, 1024) = 0.129, p =0.719, ƞ^2^= 0.0001  trait: (6, 1024) = 0.513, p = 0.799, ƞ^2^= 0.003 | N/A |
| **Rats** | *Naïve Controls and Naïve EE* | housing x trait: F(5, 542) = 0.422, p = 0.833, ƞ^2^= 0.004  housing: F(1, 542) = 0.007, p = 0.931, ƞ^2^= 0.0001  trait: F(5, 542) = 4.015, p = 0.001, ƞ^2^= 0.036 | *behavior (cns) vs anatomy*: p = 0.004 |
|  | *Treated/Manipulated Controls and Treated/Manipulated EE* | housing x trait: F(5, 696) = 0.013, p = 1.00, ƞ^2^= 0.0001  housing: F(1, 696) = 0.002, p = 0.963, ƞ^2^= 0.0001  trait: F(5, 696) = 0.460, p = 0.806, ƞ^2^= 0.003 | N/A |
| **Mice** | *Naïve Controls and Naïve EE* | housing x trait: F(6, 460) = 0.496, p = 0.811, ƞ^2^= 0.006  housing: F(1, 460) = 0.294, p = 0.588, ƞ^2^= 0.001  trait: F(6, 460) = 4.593, p = 0.0001, ƞ^2^= 0.057 | *behavior (cns) vs anatomy*: p = 0.001 |
|  | *Treated/Manipulated Controls and Treated/Manipulated EE* | housing x trait: F(6, 516) = 1.861, p = 0.086, ƞ^2^= 0.021  housing: F(1, 516) = 0.330, p = 0.566, ƞ^2^= 0.001  trait: F(6, 516) = 2.039, p = 0.059, ƞ^2^= 0.023 | N/A |
